# Validation of a Machine Learning Model to Predict Childhood Lead Poisoning

**DOI:** 10.1001/jamanetworkopen.2020.12734

**Published:** 2020-09-16

**Authors:** Eric Potash, Rayid Ghani, Joe Walsh, Emile Jorgensen, Cortland Lohff, Nik Prachand, Raed Mansour

**Affiliations:** 1 Harris School of Public Policy, University of Chicago, Chicago, Illinois; 2 Machine Learning Department, Carnegie Mellon University, Pittsburgh, Pennsylvania; 3 Chicago Department of Public Health, Chicago, Illinois; 4 Southern Nevada Health District, Las Vegas

## Abstract

**Importance:**

Childhood lead poisoning causes irreversible neurobehavioral deficits, but current practice is secondary prevention.

**Objective:**

To validate a machine learning (random forest) prediction model of elevated blood lead levels (EBLLs) by comparison with a parsimonious logistic regression.

**Design, Setting, and Participants:**

This prognostic study for temporal validation of multivariable prediction models used data from the Women, Infants, and Children (WIC) program of the Chicago Department of Public Health. Participants included a development cohort of children born from January 1, 2007, to December 31, 2012, and a validation WIC cohort born from January 1 to December 31, 2013. Blood lead levels were measured until December 31, 2018. Data were analyzed from January 1 to October 31, 2019.

**Exposures:**

Blood lead level test results; lead investigation findings; housing characteristics, permits, and violations; and demographic variables.

**Main Outcomes and Measures:**

Incident EBLL (≥6 μg/dL). Models were assessed using the area under the receiver operating characteristic curve (AUC) and confusion matrix metrics (positive predictive value, sensitivity, and specificity) at various thresholds.

**Results:**

Among 6812 children in the WIC validation cohort, 3451 (50.7%) were female, 3057 (44.9%) were Hispanic, 2804 (41.2%) were non-Hispanic Black, 458 (6.7%) were non-Hispanic White, and 442 (6.5%) were Asian (mean [SD] age, 5.5 [0.3] years). The median year of housing construction was 1919 (interquartile range, 1903-1948). Random forest AUC was 0.69 compared with 0.64 for logistic regression (difference, 0.05; 95% CI, 0.02-0.08). When predicting the 5% of children at highest risk to have EBLLs, random forest and logistic regression models had positive predictive values of 15.5% and 7.8%, respectively (difference, 7.7%; 95% CI, 3.7%-11.3%), sensitivity of 16.2% and 8.1%, respectively (difference, 8.1%; 95% CI, 3.9%-11.7%), and specificity of 95.5% and 95.1% (difference, 0.4%; 95% CI, 0.0%-0.7%).

**Conclusions and Relevance:**

The machine learning model outperformed regression in predicting childhood lead poisoning, especially in identifying children at highest risk. Such a model could be used to target the allocation of lead poisoning prevention resources to these children.

## Key Points

**Question:**

How does a machine learning model compare with logistic regression in predicting childhood lead poisoning during infancy?

**Findings:**

This prognostic study of 6812 children in a Women, Infants, and Children cohort in Chicago, Illinois, used blood lead level surveillance data, housing characteristics, and demographic characteristics to predict lead poisoning risk. Using predictors from a range of spatiotemporal scales, a random forest model significantly outperformed a parsimonious logistic regression.

**Meaning:**

These findings suggest that machine learning can be used to more precisely predict risk of elevated blood lead levels to guide allocation of prevention resources to children most at risk.

## Teaser

This prognostic study validates a machine learning (random forest) prediction model of elevated blood lead levels by comparing with a parsimonious logistic regression among children in a Women, Infants, and Children cohort.

## Introduction

Despite substantial reductions in lead hazards during the past decades, childhood lead poisoning remains a significant environmental health issue in the United States. An estimated 0.5% of children born in 2013 to 2014 have blood lead levels (BLLs) greater than the current Centers for Disease Control and Prevention reference level of 5 μg/dL (to convert to micromoles per liter, multiply by 0.0483).^1^ Elevated BLLs (EBLLs), even those below the current reference level, have been linked to neurobehavioral deficits, such as reduced intellectual and academic performance and attention-deficit/hyperactivity disorder.^2^

The main intervention that public health agencies such as the Chicago Department of Public Health (CDPH) use for lead poisoning is inspection of the home environment in cases of EBLL to identify and control hazards. This secondary prevention effort relies on reported exposure to trigger the intervention. However, no treatment has been found effective for reversing the effects of exposure.^3^ Thus the Centers for Disease Control and Prevention and American Academy of Pediatrics have called for primary prevention through proactive identification and remediation of hazards before a child develops an EBLL.^4,5^

However, primary prevention is a challenge. In Chicago, 81% of the city’s 1.2 million housing units were built before 1978, the year in which lead-based paint was banned,^6^ but few of these buildings have infants residing in them, and even fewer have reported cases of exposure. In 2017, only 1713 new EBLL (≥5 μg/dL) cases were reported, and 567 inspections were performed.

To overcome this challenge, prioritization of lead poisoning prevention resources to children most at risk of exposure has been proposed.^7,8^ A parsimonious regression model using county, median income, and housing age was developed in North Carolina.^7,9^ More recently, a machine learning model using BLL, housing, and demographic predictors at a range of spatiotemporal scales was developed for Chicago.^10^ The present study had 3 main objectives: first, to replicate the parsimonious regression model of lead poisoning using Chicago data; second, to validate the machine learning model by comparison with this regression model; and third, to pilot the targeting of lead poisoning prevention resources to children predicted to be most at risk of exposure.

## Methods

We followed the Transparent Reporting of a Multivariable Prediction Model for Individual Prognosis or Diagnosis (TRIPOD) statement for reporting multivariable prediction model validation.^11^ This work was determined to be exempt from human subjects review and informed consent by the institutional review board at the CDPH as it qualifies as an evaluation of a procedure for obtaining benefits under a public program.

### Study Population

**Figure 1.**
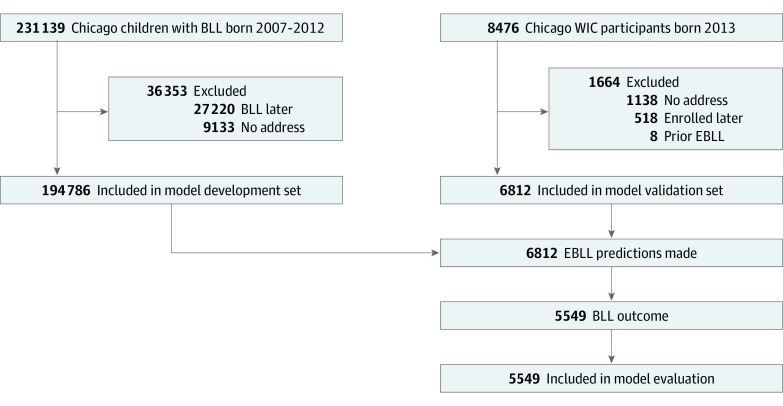
Temporal Validation Flowchart BLL indicates blood lead level; EBLL, elevated BLL; and WIC, Women, Infants, and Children.

The validation cohort or test set consisted of children enrolled in the CDPH’s Women, Infants, and Children (WIC) program and born from January 1 to December 31, 2013. The study used a temporal validation design^11^ with a split date of January 1, 2014 (Figure 1). Thus, we excluded from the test set children who enrolled in WIC after this date, who did not have an address in the WIC registry as of this date, or who had a positive outcome (see below) before this date. The development cohorts or training set consisted of all children born from January 1, 2007, to December 31, 2012, with a BLL outcome at an address in Chicago before the split date.

There were several reasons to limit the test set to WIC participants. First, to operationalize the predictions (described below), the CDPH needed current contact information, including telephone numbers, which are available for WIC participants but not for others. Second, the WIC enrollment database includes time stamps that allowed our validation to use only information that would have been available as of the split date, enhancing its validity. Finally, WIC households have lower incomes, so these children are in general at higher risk for lead poisoning.^12^ To assess how performance may change over time, we repeated this study design with validation on the 2010, 2011, and 2012 WIC cohorts.

### Outcome

The primary outcome was the binary variable of whether the child had an EBLL, defined herein as 6 μg/dL or greater in either a venous or a capillary sample. This definition was used because until 2016, some laboratories had a lower limit of detection of 5 μg/dL. In model development, the outcome was defined using BLLs measured before January 1, 2014. In validation, outcomes were measured after January 1, 2014, until December 31, 2018.

### Random Forest Model

In 2015, a random forest model was developed to predict EBLLs in Chicago children.^10^ That model was updated with additional predictors for this study. The source code is publicly available (https://github.com/chicago/lead-model). Model hyperparameters were selected using the 2010-2012 cohorts and blinded to the 2013 cohort.

#### Predictors

There were 2 types of predictors: spatial and spatiotemporal. Spatial variables were housing characteristics (age, size, and condition) from the county assessor and the Chicago building footprints shapefile (about 750 000 addresses). Spatiotemporal data sets at the address level consisted of surveillance BLLs (approximately 2.5 million tests of 1 million children since 1995); CDPH lead investigations (approximately 70 000 since 1994); Chicago Department of Buildings permits (demolition, renovation, etc) and violations (walls, windows, paint, etc; approximately 2 million since 2006); and US Census American Community Survey sociodemographic variables at the census tract level since 2005.

These data were aggregated in various ways to create predictors for the machine learning model. For example, from the historical BLLs, we calculated the EBLL (both ≥6 and ≥10 μg/dL) counts and rates at various spatial levels (address, census block, and census tract) and time periods (1, 2, 3, 5, 10, and all available years). These aggregations generated more than 1000 total predictors (eTables 1-3 in the Supplement). Predictors were developed using the development cohort data with investigators blinded to the validation cohort data.

For each data set (eg, BLLs) and aggregation level (eg, census block, 2 years), we included as a predictor the number of records in the aggregation. When equal to zero, this predictor indicated missingness of other predictors in that aggregation. Those missing values were mean imputed. Predictor importance was measured as the mean reduction in error after a tree in the forest split on that predictor.

#### Model

We used a balanced random forest model.^13,14^ Random forest uses bootstrap aggregation over observations and predictors, so we did not perform additional feature selection. The output of the model is an uncalibrated risk score from 0 to 1 for each child.

### Regression Model

For comparison, we replicated a regression model from North Carolina.^7^ Having been previously validated and deployed, this model was a natural alternative.^9^ The regression is parsimonious while including housing, economic, racial, and geographic predictors.

We made 2 changes in replication. First, because BLL outcomes in our study population are concentrated below laboratory limits of detection, we replaced the (continuous) maximum BLL outcome of the original model with the (binary) EBLL outcome. Consequently, we replaced linear with logistic regression. Second, because our analysis took place in a single county (Cook County), we replaced county fixed effects with neighborhood fixed effects. Thus, the regression generalizes the common strategy of neighborhood prioritization.^15,16^

The regression model included the following predictors: housing age from the county assessor, median income, percentage of non-Hispanic Black and Hispanic residents at the census tract level from the 5-year US Census American Community Survey, and fixed effects for 77 neighborhoods (Chicago community areas). Addresses not matching an assessor record had their age mean imputed, and a missingness indicator was included as a predictor.

### Prediction Performance Measures

Performance of the models in predicting EBLLs was summarized using the receiver operating characteristic curve and the area under the curve (AUC). More appropriate for our deployment context, we also calculated the confusion matrix metrics of positive predictive value (PPV), sensitivity, and specificity when predicting the highest-risk 5%, 10%, and 15% of children in the WIC cohort to have EBLLs. Analyses were repeated with missing outcomes imputed using a range of sensitivity parameters (eMethods in the Supplement). To investigate potential fairness concerns, metrics were calculated by race/ethnicity.^17^

### Operationalizing the Machine Learning Model to Target Proactive Inspections

On October 1, 2016, the machine learning model was updated using available data, and predictions of EBLL risk were made for children in the WIC cohort. From October 1, 2016, to September 30, 2017, the CDPH contacted WIC households to offer a proactive inspection. Offers were made to households with children younger than 12 months because these children have the greatest opportunity to benefit from a proactive inspection owing to the mean duration of 10 months it takes to complete remediation in Chicago. Households were eligible to be contacted if the child was younger than 12 months and had not had an EBLL test. The CDPH prioritized limited resources to offer inspections to children who were at higher risk and closer to the age cutoff. Predictions were updated twice (February 1 and June 1, 2017) to account for changed addresses and include newly enrolled WIC participants.

Households of children selected for inspection offers were mailed a bilingual letter (in English and Spanish) followed by a bilingual daytime telephone call offering and scheduling an inspection. The CDPH’s certified lead risk assessors used an x-ray fluorescence analyzer to complete a lead-based paint inspection per guidelines from the Department of Housing and Urban Development.^18^ They performed clearance dust wipes if no lead-based paint hazards were identified.

Inspection offers were analyzed for 2 outcomes: completion and detection of lead-based paint hazards. An estimate of the rate of lead-based paint hazards in the study population was made by reweighting estimates of significant lead-based paint hazards in the Midwest region by housing age from the American Healthy Homes Survey (AHHS).^19^^(p33)^ We also recorded the rate of lead-based paint hazards found in reactive inspections, that is, those performed by CDPH in response to an EBLL, during the same period.

### Statistical Analysis

Data were analyzed from January 1 to October 31, 2019. Statistical analyses were performed using R, version 3.3 (R Foundation for Statistical Computing) and Python, version 2.7 (Python Software Foundation) software. A 2-sided *P* ≤ .05 was considered statistically significant. We calculated 95% CIs for comparisons of AUC and confusion matrix metrics using 10 000 bootstrap replications.

## Results

Among 6812 children receiving WIC in the 2013 test set (mean [SD] age, 5.5 [0.3] years), 3451 (50.7%) were female, 3361 (49.3%) were male, 3057 (44.9%) were Hispanic, 2804 (41.2%) were non-Hispanic Black, 458 (6.7%) were non-Hispanic White, and 442 (6.5%) were Asian. The median year of housing construction was 1919 (interquartile range, 1903-1948).

Models were developed using training data for 194 786 children (Figure 1). The most important predictors in the random forest model were spatiotemporal EBLL rate aggregations (0.81-1.00); residential property values (0.52), latitude (0.47), and age (0.47); census educational level (0.30), health insurance (0.30-0.32), and income variables; lead investigation compliance (0.24), inspection (0.25-0.27), and hazard rates (0.22); and building violation counts (0.09) and the proportion pertaining to walls and windows (0.08) (Table 1). Fitted logistic regression coefficients are presented in eTable 4 in the Supplement.

**Table 1.** Most Important Predictors by Category for the Random Forest Model

Data source	Variable	Aggregation			Importance^a^	Value by outcome, mean (SD)^b^	
		Space	Years	Function		No EBLL	EBLL
Blood lead levels^c^	Child mean BLL, μg/dL	Tract	3	Median	1.00	1.4 (0.4)	1.7 (0.4)
	Child mean BLL, μg/dL	Tract	3	Mean	0.91	1.9 (0.6)	2.3 (0.6)
	Child maximum BLL, μg/dL	Tract	3	Mean	0.84	3.8 (1.1)	4.5 (1.1)
	Child EBLL ≥6 μg/dL	Tract	3	Count	0.81	16.0 (9.1)	22.0 (9.3)
	Child mean BLL, μg/dL	Tract	2	Mean	0.81	1.8 (0.5)	2.2 (0.5)
Building characteristics	Residential value, $10^5^	Block	NA	Mean	0.52	0.6 (3.5)	0.3 (1.6)
	Latitude, °	Address	NA	NA	0.47	41.9 (0.1)	41.8 (0.1)
	Housing age, y	Block	NA	Mean	0.47	80.6 (24.1)	89.1 (19.9)
	Residential value, $10^5^	Block	NA	Sum	0.47	4.8 (11.1)	3.2 (5.0)
	Rooms per unit, No.	Block	NA	Mean	0.46	5.3 (1.1)	5.2 (1.0)
American Community Survey	Medicaid insurance, No.	Tract	5	Percentage	0.32	28.6 (14.1)	34.6 (12.2)
	High school graduate, No.	Tract	5	Percentage	0.30	14.0 (7.3)	16.9 (6.9)
	Associate’s degree, No.	Tract	5	Percentage	0.30	5.0 (2.8)	4.8 (2.7)
	Employer insurance, No.	Tract	5	Percentage	0.30	41.2 (17.0)	33.9 (13.4)
	Bachelor’s degree, No.	Tract	5	Percentage	0.30	13.7 (11.6)	9.4 (8.1)
Investigations	Compliance, No.	Tract	3	Percentage	0.27	40.0 (22.6)	33.5 (18.4)
	Inspection, No.	Tract	3	Percentage	0.27	58.4 (19.6)	54.1 (16.9)
	Inspection, No.	Tract	2	Percentage	0.25	58.4 (22.0)	53.2 (19.4)
	Compliance, No.	Tract	2	Percentage	0.24	37.8 (24.8)	30.2 (20.4)
	Inspection interior hazard, No.	Tract	3	Percentage	0.22	53.8 (32.8)	62.6 (28.1)
Building permits and violations	Violations, No.	Address	All	Count	0.09	2.7 (9.2)	2.8 (8.8)
	Violations, No.	Address	5	Count	0.09	2.4 (8.3)	2.7 (8.4)
	Wall violations, No.	Address	All	Percentage	0.08	8.6 (13.4)	8.8 (11.8)
	Wall violations, No.	Address	5	Percentage	0.08	8.5 (13.5)	8.8 (11.9)
	Window violations, No.	Address	All	Percentage	0.08	6.2 (11.1)	8.0 (12.7)

Abbreviations: BLL, blood lead level; EBLL, elevated BLL; NA, not applicable.

^a^ Importance of a feature in the random forest model is measured as the mean reduction in error after a tree in the forest splits the data on that variable. Here it is rescaled to have a maximum of 1.00.

^b^ Excludes missing predictors.

^c^ Elevated levels were at least 6 μg/dL, venous or capillary samples.

**Figure 2.**
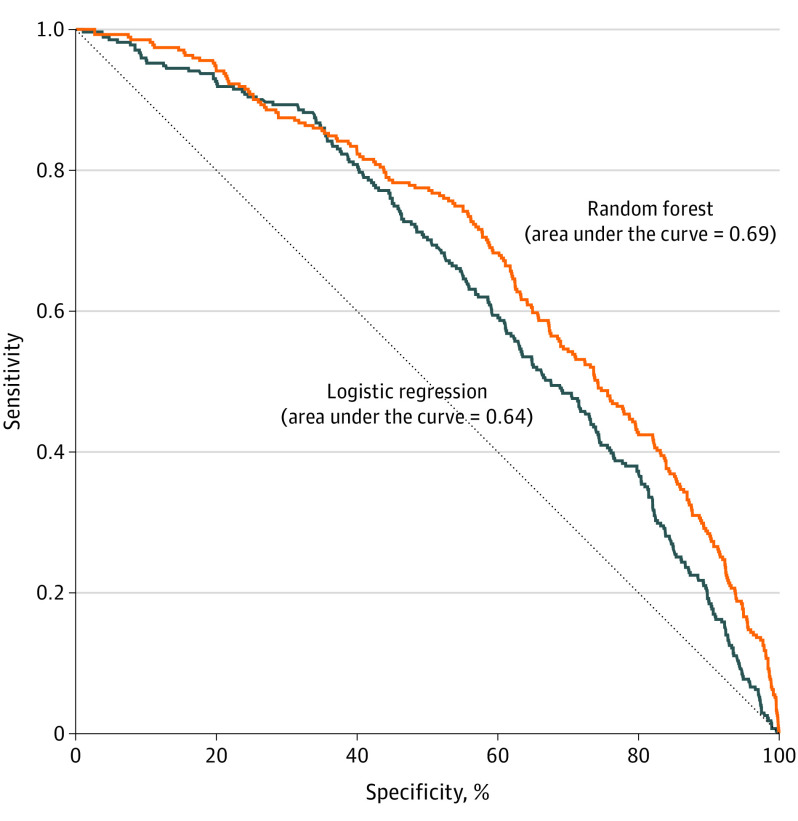
Receiver Operating Characteristic Curves for Random Forest and Logistic Regression Models Difference in the areas under the receiver operating characteristics curve was 0.05 (95% CI, 0.02-0.08).

Blood lead level outcomes were measured for 5549 children (81.4%) in the test set (Figure 1). Of these BLLs, 271 were elevated, for a prevalence of 4.9% compared with 6.8% for the training set. The AUCs of random forest and logistic regression were 0.69 and 0.64, respectively. The difference in AUC was 0.05 (95% CI, 0.02-0.08) (Figure 2).

The confusion matrix metrics for both models at thresholds of 5%, 10%, and 20% of children at highest risk are listed in Table 2. Random forest outperformed logistic regression in every metric and at every threshold. The greatest differences were in PPV and sensitivity and at higher-risk thresholds. For example, when classifying 5% of children with EBLLs, random forest and logistic regression models had PPVs of 15.5% and 7.8%, respectively (difference, 7.7%; 95% CI, 3.7%-11.3%), sensitivity of 16.2% and 8.1%, respectively (difference, 8.1%; 95% CI, 3.9%-11.7%), and specificity of 95.5% and 95.1%, respectively (difference, 0.4%; 95% CI, 0.0%-0.7%). This random forest model also significantly outperformed the originally published model specification (eTable 5 in the Supplement).

**Table 2.** Confusion Matrix Metrics for Random Forest and Logistic Regression Models

Population at highest risk, %^a^	Specificity, %			Sensitivity, %			PPV, %		
	Random forest	Logistic regression	Difference (95% CI)^b^	Random forest	Logistic regression	Difference (95% CI)^b^	Random forest	Logistic regression	Difference (95% CI)^b^
5	95.5	95.1	0.4 (0.0 to 0.7)	16.2	8.1	8.1 (3.9 to 11.7)	15.5	7.8	7.7 (3.7 to 11.3)
10	90.4	90.1	0.2 (−0.2 to 0.7)	27.3	19.9	7.4 (3.0 to 14.6)	12.7	9.4	3.3 (1.3 to 6.7)
20	80.3	79.9	0.3 (−0.1 to 1.4)	42.4	38.4	4.1 (−1.1 to 12.5)	9.9	8.9	1.0 (−0.1 to 3.0)

Abbreviation: PPV, positive predictive value.

^a^ Binary predictions are obtained from continuous risk scores by classifying this highest-risk percentage as positive.

^b^ The 95% CIs were estimated using 10 000 bootstrap replications.

Prevalence of EBLLs declined from 536 of 6337 (8.5%) in the 2010 cohort to 271 of 5549 (4.9%) in the 2013 cohort (eTable 6 in the Supplement). When repeating validation on these cohorts, some metrics also declined, for example, PPV at 5% highest risk decreased from 22.5% to 15.4%. Other metrics that are agnostic to prevalence (eg, sensitivity and AUC) were stable or increased from 2010 to 2013 (eTable 7 and eFigure in the Supplement).

In the proactive inspections pilot, offers to 478 higher-risk households yielded 27 inspections, 3 (11.1%) of which were at a different address than the risk assessment. Lead-based paint hazards were found in 20 of the 27 homes (74.1%). Using the AHHS, the estimated rate of lead-based paint hazards in the study population was 56.7% (95% CI, 46.9%-66.4%) (eTable 8 in the Supplement). Reactive inspections during the study period had an 83.8% lead hazard rate.

Blood lead levels, building characteristics, and sociodemographic predictors were rarely missing, whereas investigations and building violation and permit predictors were often missing. Missingness did not vary substantially between training and test sets (eTable 9 in the Supplement). Hispanic children were more likely both to have a BLL outcome measurement (2673 of 5585 [47.9%]) and to complete an inspection offer (12 of 27 [44.4%]). Children with no BLL outcome were predicted to have a lower mean risk by both models (eTables 10 and 11 in the Supplement). Prediction performance was robust to sensitivity analysis (eTables 12 and 13 in the Supplement). The test set of WIC participants had a higher EBLL risk compared with non-WIC participants in the same birth cohort (risk ratio, 1.4; 95% CI, 1.2-1.6) (eTable 14 in the Supplement).

## Discussion

In this prognostic study, we used machine learning to predict childhood lead exposure risk from BLL surveillance data, housing characteristics, and demographic characteristics at a range of spatiotemporal scales. We validated the predictions in the 2010-2013 Chicago WIC birth cohorts using temporal validation. We focused on the 2013 cohort validation, in which the model was fit using predictors and outcomes measured before January 1, 2014, and evaluated against outcomes occurring after the date. Performance was compared with a parsimonious regression model replicated from North Carolina.

In overall performance as measured by AUC, the machine learning model was a significant but modest improvement. However, for PPV and sensitivity at high-risk thresholds, which are the relevant metrics in our context of targeting interventions to a small proportion of the population, the improvement was significant and large.

Our study is important because both the Centers for Disease Control and Prevention and American Academy of Pediatrics have called for proactive lead poisoning interventions, but resources have not been allocated to deliver them universally.^4,5^ Targeting of lead poisoning prevention resources had been proposed as an approach to this problem.^7,8,9,10^ This study demonstrates that this approach can benefit from using machine learning methods in place of a traditional regression. Moreover, we demonstrated for the first time, to our knowledge, the use of machine learning predictions to target lead poisoning prevention interventions in practice, although uptake was low.

### Fairness, Accountability, and Transparency

The incidence of EBLL was higher among non-Hispanic Black and Hispanic participants. Prediction performance varied by race/ethnicity, with, for example, greater sensitivity and lower specificity for non-Hispanic Black participants (eTables 15-17 in the Supplement). Such variation may reflect varying risk distributions, in which case they may be considered fair.^20^ Insofar as interventions allocated using these predictions are beneficial rather than punitive, this performance could be viewed as beneficial to Black children.^21^ If, however, equal performance across race/ethnicity is preferred, this can be achieved by using different thresholds for each race, although with some limitations and to the detriment of overall performance.^22,23^

A related concern regards interpretability. Although random forest may be less interpretable than logistic regression, it is more interpretable than some other machine learning models, such as neural networks. Indeed, the most important predictors aligned with our understanding that lead poisoning is determined by age and quality of housing (Table 1). Moreover, interpretability may not be as important for this prediction application as it is for causal inference.^24,25^

### Implications

Our findings are relevant to public health and housing agencies across the country. Other municipalities may achieve better or worse results in predicting EBLLs depending on factors that include the data available to them and the prevalence of lead poisoning. Comparing results for the 2013 validation cohort with the 2010-2012 development cohorts suggests that if prevalence continues to decline into the future, absolute measures of performance (eg, PPV) may also decline, but measures that are relative to prevalence (eg, sensitivity, lift, and AUC) could remain stable or improve.

Given fixed resources for proactive interventions in Chicago, targeting using this prediction model will increase the possibility of preventing lead exposure. For example, with resources to proactively inspect 5% of children in the WIC population, targeting using random forest has the potential to prevent 16.2% of cases, twice as many as using logistic regression (8.1%). Our results suggest that lead poisoning is difficult to predict and that proactively addressing all cases would require more than the 20% intervention levels considered above. Prognosis using this predictive model is intended to supplement diagnosis using BLL testing so that secondary prevention is possible for cases that are not proactively identified for potential primary prevention.

However, successful primary prevention requires both the intervention and outreach to be effective. Regarding the intervention, a Cochrane review found that “dust control interventions may lead to little or no difference in blood lead levels” but that “the quality of evidence was moderate to low, meaning that future research is likely to change these results.”^26^^(p2)^ More recently, results of a randomized clinical trial in Cincinnati, Ohio, were reported.^27^ Overall, a comprehensive residential intervention did not result in significant BLL reductions or neurobehavioral improvements, albeit in a population with relatively low BLLs (>5 μg/dL in 3% of control group). Significant BLL reductions were, however, documented among the higher-risk non-Hispanic Black subpopulation (>5 μg/dL in 6% of control group), and the authors concluded that the “intervention may be more effective among high-risk populations.”^27^^(p941)^ Our findings show that a machine learning prediction model can identify such a high-risk subpopulation (BLL ≥ 6μg/dL in 16% of the 5% at highest risk).

The AHHS establishes that lead-based paint hazards are much more common than EBLLs. This means that when performing proactive inspections, many more hazards will be found—and thus remediations ordered—than potential EBLL cases prevented in current children occupants. Moreover, remediation is more expensive than inspection.^28^ Therefore, the efficiency of targeting proactive inspections comes not just from the targeted inspections but from the resulting targeted remediations.

Regarding outreach, uptake will likely be limited in any voluntary lead inspection program. In the Philadelphia Lead Safe Homes Study,^29^ uptake of completely subsidized lead remediation work for households of children without an EBLL was just 28%. The uptake of reactive inspections in Chicago in response to an EBLL during the study period was 58%, suggesting, unsurprisingly, that households are more likely to take up an inspection when notified by the CDPH that their child has an EBLL. Because vital statistics records do not include telephone numbers, offering proactive inspections beyond WIC is a challenge. Efforts to improve data sharing across state and local agencies may be helpful.

To use an EBLL prediction model, clinicians should first use temporal cross validation to estimate prediction performance. Next they should determine a risk threshold for intervention eligibility by examining performance (Table 2) and estimating and weighing potential health benefits and harms (such as illegal landlord retaliation) as well as costs.^28,30^ In particular, they should consider the above lack of evidence for the effectiveness of lead-hazard control in lower-risk children. Our study shows that the machine learning approach can provide substantial improvements over parsimonious regression in identifying the highest-risk children, making it effective for resource-constrained public health organizations. Clinicians should also consider factors that affect the timely completion of interventions, such as grant funding of remediation.

### Limitations

This study has several limitations. First, BLL outcomes were missing for 1263 predictions (18.5%) of the test set, and missingness was associated with race/ethnicity, although results were robust to sensitivity analyses. Outcomes were also subject to laboratory limits of detection, which were historically as high as 5 μg/dL. This prevented us from analyzing an EBLL outcome of at least 5 μg/dL.

The performance of the machine learning model, although significantly better than the regression model, leaves room for further improvement. For example, longitudinal methods can take advantage of repeated BLL measurements, and likelihood-based methods can account for measurements that were left-censored owing to lower limits of detection.^31^ Initial results suggested that alternative machine learning classifiers, such as a support vector machine, would perform similarly to the random forest model.^10^

Our estimate of the population rate of lead hazards is limited by our use of the AHHS. On the one hand, use of the AHHS may result in an overestimate, because the data were collected in 2005 to 2006, when the national EBLL (≥5 μg/dL) rate was 2.9%, and it has since declined to 0.5% in 2013 to 2014.^1^ On the other hand, it may result in an underestimate because it is based on building age alone and does not take into account the fact that the WIC population is lower income and at higher risk for lead exposure compared with the rest of the Chicago population (eTable 9 in the Supplement).

We estimated 56.7% for population, 74.1% for targeted proactive inspection, and 83.8% for reactive inspection hazard rates. The above limitations of the AHHS and the small sample size of proactive inspections limit our ability to rigorously interpret these numbers. They are, however, consistent with the hypothesis that targeted proactive inspections are about as likely to find hazards as reactive inspections, and both find hazards more often than would random inspections. Moreover, they suggest that uptake of proactive inspections was not strongly biased toward lower-risk households.

Mobility and/or poor data quality resulted in 3 of 27 targeted proactive inspections (11.1%) being scheduled at a different address from where the child was predicted to be at risk. Mobility presents 2 limitations. First, a child’s risk may change between prediction and intervention. Second, if a child moves after a residential intervention, they are not likely to fully benefit.

## Conclusions

Our study provides evidence that machine learning can improve on parsimonious regression in predicting the risk for childhood lead poisoning. We also demonstrated the feasibility of using model predictions to target proactive residential inspections for lead, potentially enabling a shift from secondary to primary prevention. However, uptake of voluntary inspection offers was low, and evidence of their effectiveness is currently incomplete. We are implementing this system to provide predictions to physicians, community health workers, and community-based organizations so that resources for lead poisoning prevention may be allocated to children most at risk of exposure.

## Notes

Supplement. **eTable 1.** Spatiotemporal Predictors in the Random Forest Model **eTable 2.** Spatial Predictors in the Random Forest Model **eTable 3.** Sociodemographic Predictors in the Random Forest Model **eTable 4.** Fitted Coefficients for the Logistic Regression Model **eTable 5.** Comparison Between Original and Updated Random Forest Models of Confusion Matrix Metrics on 2013 Cohort **eTable 6.** Elevated Blood Lead Level Outcomes by Validation Cohort **eTable 7.** Confusion Matrix Metrics for the Random Forest Model by Validation Cohort **eTable 8.** Inspection Cohort Lead Hazard Rate Estimate **eTable 9.** Comparison of Most Important Random Forest Predictor Values in Training and Test Sets **eTable 10.** Baseline Characteristics of 2013 Cohort, With and Without Measured Outcome **eTable 11.** Baseline Characteristics of Inspection Cohort, Inspected and Uninspected **eTable 12.** Sensitivity Analysis of Area Under Receiver Operating Characteristic Curve **eTable 13.** Sensitivity Analysis of Confusion Matrix Metrics **eTable 14.** Elevated Blood Lead Level Risk in 2013 Cohort by Enrollment in Women, Infants, and Children (WIC) Program **eTable 15.** Elevated Blood Lead Level Risk in 2013 Cohort by Race/Ethnicity **eTable 16.** Race/Ethnicity by Risk Group for the Random Forest Model **eTable 17.** Confusion Matrix Metrics for the Random Forest Model by Race/Ethnicity **eMethods.** Sensitivity Analysis **eFigure.** Receiver Operating Characteristic Curves by Validation Cohort

## References

[1] Tsoi M-F, Cheung C-L, Cheung TT, Cheung BMY Continual decrease in blood lead level in Americans: United States National Health Nutrition and Examination Survey 1999-2014. Am J Med. 2016;129(11):1213-1218. doi:10.1016/j.amjmed.2016.05.042 27341956

[2] National Toxicology Program NTP monograph on health effects of low-level lead. NTP Monogr. 2012;(1):e2012734. 23964424

[3] Dietrich KN, Ware JH, Salganik M, ; Treatment of Lead-Exposed Children Clinical Trial Group Effect of chelation therapy on the neuropsychological and behavioral development of lead-exposed children after school entry. Pediatrics. 2004;114(1):19-26. doi:10.1542/peds.114.1.19 15231903

[4] Centers for Disease Control and Prevention Low level lead exposure harms children: a renewed call of primary prevention: report of the Advisory Committee on Childhood Lead Poisoning Prevention. Published January 4, 2012. Accessed October 31, 2019. https://www.cdc.gov/nceh/lead/acclpp/final_document_030712.pdf

[5] Council on Environmental Health Prevention of childhood lead toxicity. Pediatrics. 2016;138(1):e20161493. doi:10.1542/peds.2016-1493 27325637

[6] Fokum F, Simpson E, McAffee K Illinois Lead Program 2015 Annual Surveillance Report. December 2016 Accessed October 31, 2019. http://dph.illinois.gov/sites/default/files/publications/lead-surveillance-report-2015-122116.pdf

[7] Miranda ML, Dolinoy DC, Overstreet MA Mapping for prevention: GIS models for directing childhood lead poisoning prevention programs. Environ Health Perspect. 2002;110(9):947-953. doi:10.1289/ehp.02110947 12204831 PMC1240996

[8] Lanphear BP, Hornung R, Ho M Screening housing to prevent lead toxicity in children. Public Health Rep. 2005;120(3):305-310. doi:10.1177/003335490512000315 16134573 PMC1497723

[9] Kim D, Galeano MAO, Hull A, Miranda ML A framework for widespread replication of a highly spatially resolved childhood lead exposure risk model. Environ Health Perspect. 2008;116(12):1735-1739. doi:10.1289/ehp.11540 19079729 PMC2599772

[10] Potash E, Brew J, Loewi A, Predictive modeling for public health: preventing childhood lead poisoning. In: Proceedings of the 21st ACM SIGKDD International Conference on Knowledge Discovery and Data Mining. Association for Computing Machinery; 2015:2039-2047. doi:10.1145/2783258.2788629

[11] Moons KGM, Altman DG, Reitsma JB, Transparent Reporting of a Multivariable Prediction Model for Individual Prognosis or Diagnosis (TRIPOD): explanation and elaboration. Ann Intern Med. 2015;162(1):W1-73. doi:10.7326/M14-0698 25560730

[12] Centers for Disease Control and Prevention Childhood lead poisoning prevention: at-risk populations. Reviewed July 30, 2019. Accessed October 31, 2019. https://www.cdc.gov/nceh/lead/prevention/populations.htm

[13] Breiman L Random forests. Mach Learn. 2001;45:5-32. doi:10.1023/A:1010933404324

[14] Chen C, Liaw A, Breiman L Using random forest to learn imbalanced data. Published July 2004. Accessed October 31, 2019. https://statistics.berkeley.edu/sites/default/files/tech-reports/666.pdf

[15] Meyer PA, Staley F, Staley P, Curtis J, Blanton C, Brown MJ Improving strategies to prevent childhood lead poisoning using local data. Int J Hyg Environ Health. 2005;208(1-2):15-20. doi:10.1016/j.ijheh.2005.01.003 15881974

[16] Dignam TA, Evens A, Eduardo E, High-intensity targeted screening for elevated blood lead levels among children in 2 inner-city Chicago communities. Am J Public Health. 2004;94(11):1945-1951. doi:10.2105/AJPH.94.11.1945 15514235 PMC1448567

[17] Mitchell S, Potash E, Barocas S, D’Amour A, Lum K Prediction-based decisions and fairness: a catalogue of choices, assumptions, and definitions. Published April 24, 2020. Accessed October 31, 2019. https://arxiv.org/abs/1811.07867v3

[18] US Department of Housing and Urban Development Guidelines for the evaluation and control of lead-based paint hazards in housing. Published July 2012 Accessed October 31, 2019. https://www.hud.gov/program_offices/healthy_homes/lbp/hudguidelines

[19] US Department of Housing and Urban Development American Healthy Homes Survey: lead and arsenic findings. Published April 2011 Accessed October 31, 2019. https://www.hud.gov/sites/documents/AHHS_REPORT.PDF

[20] Corbett-Davies S, Pierson E, Feller A, Goel S, Huq A Algorithmic decision making and the cost of fairness. In: Proceedings of the 23rd ACM SIGKDD International Conference on Knowledge Discovery and Data Mining. Association for Computing Machinery; August 2017:797-806. doi:10.1145/3097983.3098095

[21] Saleiro P, Kuester B, Hinkson L, Aequitas: a bias and fairness audit toolkit. April 29, 2019. Accessed October 31, 2019. https://arxiv.org/abs/1811.05577

[22] Hardt M, Price E, Srebro N Equality of opportunity in supervised learning. In: Lee DD, Sugiyama M, Luxburg UV, Guyon I, Garnett R. Advances in Neural Information Processing Systems. Curran Associates; 2016.

[23] Chouldechova A Fair prediction with disparate impact: a study of bias in recidivism prediction instruments. Big Data. 2017;5(2):153-163. doi:10.1089/big.2016.0047 28632438

[24] Kleinberg J, Ludwig J, Mullainathan S, Obermeyer Z Prediction policy problems. Am Econ Rev. 2015;105(5):491-495. doi:10.1257/aer.p20151023 27199498 PMC4869349

[25] Lipton ZC The mythos of model interpretability. Queue. 2018;61(10):36-43. doi:10.1145/3233231

[26] Nussbaumer-Streit B, Yeoh B, Griebler U, Household interventions for preventing domestic lead exposure in children. Cochrane Database Syst Rev. 2016;10:CD006047. doi:10.1002/14651858.CD006047.pub5 27744650 PMC6461195

[27] Braun JM, Hornung R, Chen A, Effect of residential lead-hazard interventions on childhood blood lead concentrations and neurobehavioral outcomes: a randomized clinical trial. JAMA Pediatr. 2018;172(10):934-942. doi:10.1001/jamapediatrics.2018.2382 30178064 PMC6233767

[28] Gould E Childhood lead poisoning: conservative estimates of the social and economic benefits of lead hazard control. Environ Health Perspect. 2009;117(7):1162-1167. doi:10.1289/ehp.0800408 19654928 PMC2717145

[29] Campbell C, Tran M, Gracely E, Primary prevention of lead exposure: the Philadelphia Lead Safe Homes Study. Public Health Rep. 2011;126(suppl 1):76-88. doi:10.1177/00333549111260S111 21563715 PMC3072906

[30] Billings SB, Schnepel KT Life after lead: effects of early interventions for children exposed to lead. Am Econ J Appl Econ. 2018;10(3):315-344. doi:10.1257/app.20160056

[31] Fitzmaurice GM, Laird NM, Ware JH Applied Longitudinal Analysis. 2nd ed John Wiley & Sons; 2011. doi:10.1002/9781119513469

