# Supplementary material for: Validation of a Machine Learning Model to Predict Childhood Lead Poisoning
Source: JAMA Netw Open. 2020 Sep 16;3(9):e2012734. doi: 10.1001/jamanetworkopen.2020.12734 (PMC7495240; doi:10.1001/jamanetworkopen.2020.12734)
Supplement: Supplement. — eTable 1. Spatiotemporal Predictors in the Random Forest Model eTable 2. Spatial Predictors in the Random Forest Model eTable 3. Sociodemographic Predictors in the Random Forest Model eTable 4. Fitted Coefficients for the Logistic Regression Model eTable 5. Comparison Between Original and Updated Random Forest Models of Confusion Matrix Metrics on 2013 Cohort eTable 6. Elevated Blood Lead Level Outcomes by Validation Cohort eTable 7. Confusion Matrix Metrics for the Random Forest Model by Validation Cohort eTable 8. Inspection Cohort Lead Hazard Rate Estimate eTable 9. Comparison of Most Important Random Forest Predictor Values in Training and Test Sets eTable 10. Baseline Characteristics of 2013 Cohort, With and Without Measured Outcome eTable 11. Baseline Characteristics of Inspection Cohort, Inspected and Uninspected eTable 12. Sensitivity Analysis of Area Under Receiver Operating Characteristic Curve eTable 13. Sensitivity Analysis of Confusion Matrix Metrics eTable 14. Elevated Blood Lead Level Risk in 2013 Cohort by Enrollment in Women, Infants, and Children (WIC) Program eTable 15. Elevated Blood Lead Level Risk in 2013 Cohort by Race/Ethnicity eTable 16. Race/Ethnicity by Risk Group for the Random Forest Model eTable 17. Confusion Matrix Metrics for the Random Forest Model by Race/Ethnicity eMethods. Sensitivity Analysis eFigure. Receiver Operating Characteristic Curves by Validation Cohort [file jamanetwopen-e2012734-s001.pdf]

Supplementary Online Content

Potash E, Ghani R, Walsh J, et al. Validation of a machine learning model to predict childhood lead poisoning. *JAMA Netw Open*. 2020;3(9):e2012734. doi:10.1001/jamanetworkopen.2020.12734

eTable 1. Spatiotemporal Predictors in the Random Forest Model

eTable 2. Spatial Predictors in the Random Forest Model

eTable 3. Sociodemographic Predictors in the Random Forest Model

eTable 4. Fitted Coefficients for the Logistic Regression Model

eTable 5. Comparison Between Original and Updated Random Forest Models of Confusion Matrix Metrics on 2013 Cohort

eTable 6. Elevated Blood Lead Level Outcomes by Validation Cohort

eTable 7. Confusion Matrix Metrics for the Random Forest Model by Validation Cohort

eTable 8. Inspection Cohort Lead Hazard Rate Estimate

eTable 9. Comparison of Most Important Random Forest Predictor Values in Training and Test Sets

eTable 10. Baseline Characteristics of 2013 Cohort, With and Without Measured Outcome

eTable 11. Baseline Characteristics of Inspection Cohort, Inspected and Uninspected

eTable 12. Sensitivity Analysis of Area Under Receiver Operating Characteristic Curve

eTable 13. Sensitivity Analysis of Confusion Matrix Metrics

eTable 14. Elevated Blood Lead Level Risk in 2013 Cohort by Enrollment in Women, Infants, and Children (WIC) Program

eTable 15. Elevated Blood Lead Level Risk in 2013 Cohort by Race/Ethnicity

eTable 16. Race/Ethnicity by Risk Group for the Random Forest Model

eTable 17. Confusion Matrix Metrics for the Random Forest Model by Race/Ethnicity

eMethods. Sensitivity Analysis

eFigure. Receiver Operating Characteristic Curves by Validation Cohort

This supplementary material has been provided by the authors to give readers additional information about their work.

©2020 Potash E et al. *JAMA Network Open*.

eTable 1. Spatiotemporal Predictors in the Random Forest Model

Data Source	Variable	Aggregation Functions
Blood Lead Levels	Address days between first and last sample	mean
	Address days since BLL sample	max, min
	Address days since EBLL $\geq$ 10 µg/dL	max, min
	Address days since EBLL $\geq$ 6 µg/dL	max, min
	Child age	max, mean, min
	Child average BLL	max, mean, median, min
	Child maximum BLL	max, mean, median, min
	Child EBLL $\geq$ 10 µg/dL	count, rate
	Child EBLL $\geq$ 10 µg/dL at present address	count, rate
	Child EBLL $\geq$ 10 µg/dL at previous address	count, rate
	Child EBLL $\geq$ 10 µg/dL at subsequent address	count, rate
	Child EBLL $\geq$ 6 µg/dL	count, rate
	Child EBLL $\geq$ 6 µg/dL at present address	count, rate
	Child EBLL $\geq$ 6 µg/dL at previous address	count, rate
	Child EBLL $\geq$ 6 µg/dL at subsequent address	count, rate
	Child number of BLL samples	max, mean
	Child number of addresses	max, mean
	Child screened	%, count
Building Permits and Violations	Complied	%, count
	Easy permits	%, count
	Electric wiring permits	%, count
	Elevator permits	%, count
	Extension permits	%, count
	New construction permits	%, count
	No entry	%, count
	Open violation	%, count
	Paint violations	%, count
	Permits	count
	Porch permits	%, count
	Porch violations	%, count
	Reinstate permits	%, count
	Renovation/alteration permits	%, count
	Scaffolding permits	%, count
	Signs permits	%, count
	Violations	count
	Wall violations	%, count
	Water violations	%, count
	Window violations	%, count
	Wrecking/demolition permits	%, count
Investigations	Compliance	%, count
	Days since case closure	max, mean, min
	Days since compliance	max, mean, min
	Days since inspection	max, mean, min

©2020 Potash E et al. *JAMA Network Open*.

Data Source	Variable	Aggregation Functions
	Days since referral	max, mean, min
	Inspection	%, count
	Inspection Interior or exterior hazard	count
	Inspection exterior hazard	count
	Inspection interior and exterior hazard	count
	Inspection interior hazard	count
	Investigation count	%, count
	Open investigation	%, count

Each variable is aggregated using the specified functions at the following 11 spatial levels and time periods: at the address level over 1, 2, 5, and 10, and all available years; at the block level over 1, 2, and 5 years; and at the tract level over 1, 2 and 3 years. Abbrevations: EBLL, elevated blood lead level.

©2020 Potash E et al. *JAMA Network Open*.

eTable 2. Spatial Predictors in the Random Forest Model

Variable	Aggregation Functions
Assessed brownfield properties	%
Assessed commercial properties	%
Assessed incentive properties	%
Assessed industrial properties	%
Assessed multifamily properties	%
Assessed nonprofit properties	%
Assessed properties	count
Assessed residential properties	%, count
Baths per unit	mean
Beds per unit	mean
Building area	sum
Building footprints	count
Building needs major repair	%, any
Building needs minor repair	%, any
Building sound	%, any
Building uninhabitable	%, any
Building volume	mean
Land value	mean
Maximum housing age	max, mean, min
Minimum housing age	max, mean, min
Non-residential value (10^5$)	mean, sum
Number of addresses	sum
Number of apartments	mean
Number of assessments	mean
Number of non-residential units	count
Number of stories	mean
Number of units	mean, sum
Owner-occupied	%
Residential value (10^5$)	mean, sum
Rooms per unit	mean
Year built before 1978	%
Year built	max, mean, median, min

Each variable is aggregated using the specified functions at the following spatial scales: address, building, complex, block, tract.

©2020 Potash E et al. *JAMA Network Open*.

eTable 3. Sociodemographic Predictors in the Random Forest Model

Category	Variable
Educational Attainment of Adults	Less than 9th grade
	9th to 12th grade, no diploma
	High school graduate (includes equivalency)
	Some college, no degree
	Associate's degree
	Bachelor's degree
	Graduate or professional degree
Educational Attainment of Recent Mothers	Less than high school graduate
	High school graduate (includes equivalency)
	Some college or associate's degree
	Bachelor's degree
	Graduate or professional degree
Family Poverty Status	Below poverty level
Health Insurance	Uninsured
	Employer
	Purchased
	Medicare
	Medicaid
	Military
	Veteran
Housing Unit Tenure	Owner-occupied
Labor Force Participation of Recent Mothers	In labor force
Marital Status of Recent Mothers	Married
Median Income	Median income
Poverty Status of Recent Mothers	Below 100% of poverty level
	100 and 199% of poverty level
	200% or more of poverty level
Public Assistance Status of Recent Mothers	Receiving public assistance
Race/Ethnicity	Asian
	Black
	White
	Hispanic

All variables are from the American Community Survey and at the tract level. All variables except median income are calculated as proportions within the relevant population.

©2020 Potash E et al. *JAMA Network Open*.

eTable 4. Fitted Coefficients for the Logistic Regression Model

Predictor	Coefficient	SE
Tract Median Income	-2.39×10^{-5}	1.45×10^{-6}
Tract Percentage Black	0.232	0.075
Tract Percentage Hispanic	-0.013	0.077
Assessor Age Present	-0.066	0.019
Assessor Age	7.74×10^{-3}	3.17×10^{-4}
Albany Park	-2.854	0.085
Archer Heights	-2.683	0.121
Armour Square	-3.185	0.149
Ashburn	-2.744	0.115
Auburn Gresham	-2.415	0.100
Austin	-2.296	0.090
Avalon Park	-2.540	0.155
Avondale	-2.748	0.095
Belmont Cragin	-2.771	0.087
Beverly	-2.407	0.164
Bridgeport	-2.993	0.098
Brighton Park	-2.285	0.088
Burnside	-2.541	0.209
Calumet Heights	-2.704	0.159
Chatham	-2.697	0.108
Chicago Lawn	-2.283	0.091
Clearing	-2.957	0.142
Douglas	-3.281	0.142
Dunning	-3.314	0.137
East Garfield Park	-2.593	0.104
East Side	-2.641	0.109
Edgewater	-2.800	0.098
Edison Park	-3.148	0.299
Englewood	-2.308	0.095
Forest Glen	-3.445	0.261
Fuller Park	-2.453	0.179
Gage Park	-2.277	0.093
Garfield Ridge	-3.051	0.128
Grand Boulevard	-2.731	0.113
Greater Grand Crossing	-2.421	0.099
Hegewisch	-2.665	0.174
Hermosa	-2.638	0.106
Humboldt Park	-2.378	0.091
Hyde Park	-3.087	0.139
Irving Park	-2.840	0.093
Jefferson Park	-3.175	0.155
Kenwood	-2.824	0.137
Lake View	-3.382	0.143
Lincoln Park	-3.345	0.166
Lincoln Square	-2.928	0.111
Logan Square	-2.699	0.087
Loop	-3.196	0.251
Lower West Side	-2.571	0.095
Mckinley Park	-2.473	0.103
Montclare	-3.160	0.165
Morgan Park	-2.730	0.136

©2020 Potash E et al. *JAMA Network Open*.

Predictor	Coefficient	SE
Mount Greenwood	-3.850	0.343
Near North Side	-3.047	0.126
Near South Side	-3.058	0.199
Near West Side	-2.996	0.108
New City	-1.992	0.086
North Center	-3.147	0.153
North Lawndale	-2.373	0.093
North Park	-3.080	0.151
Norwood Park	-3.347	0.178
Oakland	-3.533	0.213
Ohare	-2.873	0.199
Portage Park	-2.947	0.090
Pullman	-2.576	0.159
Riverdale	-3.674	0.307
Rogers Park	-2.889	0.088
Roseland	-2.303	0.100
South Chicago	-2.259	0.096
South Deering	-3.003	0.130
South Lawndale	-2.137	0.091
South Shore	-2.715	0.097
Uptown	-3.005	0.094
Washington Heights	-2.384	0.118
Washington Park	-2.820	0.113
West Elsdon	-2.722	0.116
West Englewood	-2.244	0.094
West Garfield Park	-2.358	0.103
West Lawn	-2.786	0.104
West Pullman	-2.357	0.104
West Ridge	-2.727	0.072
West Town	-2.850	0.096
Woodlawn	-2.521	0.104

©2020 Potash E et al. *JAMA Network Open*.

eTable 5. Comparison Between Original and Updated Random Forest Models of Confusion Matrix Metrics on 2013 Cohort

	Specificity			Sensitivity			PPV		
	**Updated**	**Original**	**Difference**	**Updated**	**Original**	**Difference**	**Updated**	**Original**	**Difference**
**Highest-risk %[a]**									
0.05	95.5%	95.3%	0.1% (-0.2% to 0.4%)	16.2%	10.7%	5.5% (0.8% to 9.2%)	15.5%	10.5%	4.9% (0.5% to 8.5%)
0.10	90.4%	90.4%	-0.0% (-0.4% to 0.3%)	27.3%	21.8%	5.5% (1.4% to 11.1%)	12.7%	10.4%	2.3% (0.3% to 4.8%)
0.20	80.3%	80.1%	0.2% (-0.3% to 0.6%)	42.4%	34.7%	7.7% (2.9% to 13.3%)	9.9%	8.2%	1.7% (0.6% to 3.1%)

Confidence intervals estimated using 10 000 bootstrap replications. Abbreviations: PPV, positive predictive value

[a] Binary predictions are obtained from continuous risk scores by classifying this highest-risk percentage as positive.

©2020 Potash E et al. *JAMA Network Open*.

eTable 6. Elevated Blood Lead Level Outcomes by Validation Cohort

Cohort Year	Cohort Size	BLL outcome measured (%)	EBLL outcome (%)
2010	7524	6337 (84.2%)	536 (8.5%)
2011	7360	6127 (83.2%)	439 (7.2%)
2012	7205	5949 (82.6%)	345 (5.8%)
2013	6812	5549 (81.5%)	271 (4.9%)

Abbreviations: BLL, blood lead level (venous or capillary); EBLL, elevated blood lead level ≥ 6μg/dL (venous or capillary).

©2020 Potash E et al. *JAMA Network Open*.

eTable 7. Confusion Matrix Metrics by Validation Cohort

2010

	Specificity			Sensitivity			PPV		
	RF	LR	Difference	RF	LR	Difference	RF	LR	Difference
**Highest-risk %[a]**									
5%	95.8%	95.2%	0.6% (0.3% to 1.0%)	13.2%	8.6%	4.7% (2.0% to 7.9%)	22.5%	14.1%	8.5% (4.0% to 13.9%)
10%	91.1%	90.4%	0.7% (0.2% to 1.2%)	22.9%	17.0%	6.0% (2.1% to 9.4%)	19.2%	14.0%	5.2% (2.2% to 8.1%)
20%	81.2%	80.5%	0.7% (0.2% to 1.3%)	37.1%	31.9%	5.2% (1.1% to 8.9%)	15.4%	13.1%	2.3% (0.7% to 3.9%)

2011

	Specificity			Sensitivity			PPV		
	RF	LR	Difference	RF	LR	Difference	RF	LR	Difference
**Highest-risk %[a]**									
5%	95.4%	95.0%	0.5% (0.1% to 0.8%)	13.9%	9.6%	4.3% (0.9% to 7.6%)	19.1%	12.8%	6.2% (1.6% to 10.5%)
10%	90.8%	89.9%	0.8% (0.4% to 1.3%)	24.6%	18.0%	6.6% (2.2% to 11.1%)	17.1%	12.1%	5.0% (2.0% to 7.7%)
20%	80.9%	80.1%	0.8% (0.4% to 1.5%)	40.5%	37.1%	3.4% (-1.2% to 8.4%)	14.1%	12.6%	1.5% (0.0% to 3.2%)

2012

	Specificity			Sensitivity			PPV		
	RF	LR	Difference	RF	LR	Difference	RF	LR	Difference
**Highest-risk %[a]**									
5%	95.5%	94.9%	0.6% (0.2% to 0.8%)	15.4%	9.9%	5.5% (0.9% to 9.2%)	17.3%	10.7%	6.6% (1.6% to 10.3%)
10%	90.7%	89.9%	0.8% (0.3% to 1.2%)	26.7%	19.4%	7.2% (2.6% to 11.3%)	15.0%	10.6%	4.4% (1.7% to 6.6%)
20%	80.6%	79.9%	0.7% (0.1% to 1.1%)	40.3%	38.3%	2.0% (-3.1% to 7.3%)	11.4%	10.5%	0.9% (-0.6% to 2.3%)

2013

	Specificity			Sensitivity			PPV		
	RF	LR	Difference	RF	LR	Difference	RF	LR	Difference
**Highest-risk %[a]**									
5%	95.5%	95.1%	0.4% (0.0% to 0.7%)	16.2%	8.1%	8.1% (3.9% to 11.7%)	15.5%	7.8%	7.7% (3.7% to 11.3%)
10%	90.4%	90.1%	0.2% (-0.2% to 0.7%)	27.3%	19.9%	7.4% (3.0% to 14.6%)	12.7%	9.4%	3.3% (1.3% to 6.7%)
20%	80.3%	79.9%	0.3% (-0.1% to 1.4%)	42.4%	38.4%	4.1% (-1.1% to 12.5%)	9.9%	8.9%	1.0% (-0.1% to 3.0%)

Confidence intervals estimated using 10 000 bootstrap replications. Abbreviations: PPV, positive predictive value; RF, random forest; LR, logistic regression

[a] Binary predictions are obtained from continuous risk scores by classifying this highest-risk percentage as positive.

©2020 Potash E et al. *JAMA Network Open*.

eTable 8. Inspection Cohort Lead Hazard Rate Estimate

Housing Age	Number (Proportion)[a]	Hazard Rate (95% CI)[b]
Before 1940	6678 (73.8%)	68.5% (58.9% to 78.0%)
1940-1959	993 (11.0%)	48.7% (30.3% to 67.1%)
1960-1977	816 (9.0%)	7.7% (1.3% to 14.1%)
After 1978	563 (6.2%)	1.2% (0.0% to 2.7%)
Total	9050 (100%)	56.7% (46.9% to 66.4%)[c]

[a] Excludes 880 homes with missing age
[b] From Midwest estimates of American Healthy Homes Survey Table 5-1
[c] Housing age assumed to be missing at random

©2020 Potash E et al. *JAMA Network Open*.

eTable 9. Comparison of Most Important Predictor Characteristics in Training and Test Sets

Aggregation			Variable	Training Set		Test Set	
**Space**	**Function**	**Years**		**Mean (SD)**	**Missing (%)**	**Mean (SD)**	**Missing (%)**
Tract	median	3	Child Average BLL (μg/dL)	1.1 (0.2)	0.0%	1.5 (0.4)	0.1%
Tract	mean	3	Child Average BLL (μg/dL)	1.5 (0.3)	0.0%	2.0 (0.6)	0.1%
Tract	mean	3	Maximum BLL (μg/dL)	2.9 (0.6)	0.0%	3.9 (1.2)	0.1%
Tract	count	3	Child EBLL ≥ 6 μg/dL	9.2 (4.7)	0.0%	16.7 (9.3)	0.1%
Tract	mean	2	Child Average BLL (μg/dL)	1.4 (0.3)	0.0%	1.9 (0.5)	0.1%
Block	units	--	Residential value (10^5\$)	0.6 (3.2)	2.2%	0.5 (3.4)	2.4%
Address		--	Latitude (°)	41.8 (0.1)	0.0%	41.9 (0.1)	0.0%
Block	mean	--	Minimum Housing Age	83.0 (24.2)	1.7%	81.3 (23.9)	2.0%
Block	value	--	Residential value (10^5\$)	4.0 (6.4)	2.2%	4.7 (10.9)	2.4%
Block	mean	--	Rooms per unit	5.2 (1.0)	10.2%	5.3 (1.1)	9.4%
Tract	%	5	Medicaid insurance	31.7 (13.3)	0.0%	29.0 (14.1)	0.1%
Tract	%	5	High school education	14.5 (6.8)	0.0%	14.3 (7.3)	0.1%
Tract	%	5	Associate's degree	5.2 (2.5)	0.0%	5.0 (2.8)	0.1%
Tract	%	5	Employer insurance	36.9 (14.9)	0.0%	40.7 (16.9)	0.1%
Tract	%	5	Bachelors degree	11.8 (9.8)	0.0%	13.4 (11.4)	0.1%
Tract	%	3	Compliance	38.4 (21.0)	16.4%	39.4 (22.3)	12.9%
Tract	%	3	Inspection	57.3 (19.9)	10.4%	58.0 (19.4)	8.5%
Tract	%	2	Inspection	55.6 (21.5)	16.4%	58.0 (21.8)	13.8%
Tract	%	2	Compliance	35.0 (21.8)	27.8%	37.1 (24.6)	24.6%
Tract	count	3	Inspection Interior Hazard	62.9 (31.1)	10.5%	54.4 (32.5)	8.7%
Address	count	all	Violations	5.5 (14.9)	0.0%	2.7 (9.0)	0.0%
Address	count	5	Violations	3.2 (9.5)	0.0%	2.4 (8.3)	0.0%
Address	%	all	Wall violations	8.7 (12.2)	68.8%	8.6 (13.3)	79.4%
Address	%	5	Wall violations	8.5 (13.0)	76.4%	8.5 (13.4)	80.4%
Address	%	all	Window violations	6.4 (10.0)	68.8%	6.3 (11.3)	79.4%

©2020 Potash E et al. *JAMA Network Open*.

eTable 10. Baseline Characteristics of 2013 Cohort, With and Without a Measured BLL Outcome

Covariate	No BLL outcome (n=1263)	BLL outcome (n=5549)
Female	621 (49%)	2830 (51%)
Race/ethnicity		
Hispanic	420 (33%)	2637 (48%)
Non-Hispanic Black	514 (41%)	2290 (41%)
Non-Hispanic White	184 (15%)	274 (5%)
Asian	122 (10%)	320 (6%)
Other	23 (2%)	28 (1%)
Housing age		
Before 1940	683 (54%)	3546 (64%)
1940-1959	133 (11%)	492 (9%)
1960-1977	109 (9%)	398 (7%)
After 1977	106 (8%)	320 (6%)
Missing	232 (18%)	793 (14%)
RF Risk score (IQR)	0.19 (0.15 to 0.25)	0.21 (0.16 to 0.27)
LR Risk score (IQR)	0.07 (0.05 to 0.12)	0.10 (0.06 to 0.13)

Abbreviations: BLL, blood lead level (venous or capillary); RF, random forest; LR, logistic regression.

©2020 Potash E et al. *JAMA Network Open*.

eTable 11. Baseline Characteristics of Inspection Cohort, Inspected and Uninspected

**Covariate**	**Uninspected (n=451)**	**Inspected (n=27)**
Female	248 (55%)	14 (52%)
Race/ethnicity		
Hispanic	158 (35%)	12 (44%)
Non-Hispanic Black	253 (56%)	13 (48%)
Non-Hispanic White	17 (4%)	1 (4%)
Asian	19 (4%)	1 (4%)
Other	4 (1%)	0 (0%)
Housing age		
Before 1940	407 (94%)	24 (96%)
1940-1959	15 (3%)	1 (4%)
1960-1977	6 (1%)	0 (0%)
After 1977	3 (1%)	0 (0%)
Missing	20 (4%)	2 (7%)
Risk score (IQR)	0.43 (0.40 to 0.49)	0.42 (0.40 to 0.46)

©2020 Potash E et al. *JAMA Network Open*.

eTable 12. Sensitivity Analysis of Area Under Receiver Operating Characteristic Curve

	AUC		
δ	**RF**	**LR**	**Difference**
-0.25	0.70	0.66	.04 (.02 to .07)
+0.25	0.68	0.63	.05 (.02 to .07)

Abbreviations: AUC, Area Under Receiver Operating Characteristic Curve; RF, random forest; LR, logistic regression

©2020 Potash E et al. *JAMA Network Open*.

eTable 13. Sensitivity Analysis of Confusion Matrix Metrics

δ=-25%

	Specificity			Sensitivity			PPV		
	RF	LR	Difference	RF	LR	Difference	RF	LR	Difference
Highest-risk %[a]									
5%	95.5%	95.1%	0.4% (0.2% to 0.6%)	16.7%	8.4%	8.3% (3.0% to 12.7%)	15.2%	7.6%	7.6% (2.8% to 11.7%)
10%	90.8%	90.4%	0.4% (0.2% to 0.7%)	27.3%	18.9%	8.4% (3.6% to 13.9%)	12.5%	8.7%	3.8% (1.2% to 6.2%)
20%	81.1%	80.6%	0.5% (0.0% to 1.1%)	42.5%	36.5%	6.1% (-2.0% to 12.5%)	9.7%	8.3%	1.5% (-0.3% to 3.1%)

δ=+25%

	Specificity			Sensitivity			PPV		
	RF	LR	Difference	RF	LR	Difference	RF	LR	Difference
Highest-risk %[a]									
5%	95.6%	95.2%	0.4% (0.1% to 0.7%)	16.6%	8.9%	7.7% (2.2% to 11.9%)	16.7%	8.8%	7.8% (1.6% to 12.0%)
10%	90.9%	90.5%	0.4% (0.1% to 0.7%)	27.6%	19.4%	8.2% (1.6% to 14.1%)	13.8%	9.7%	4.1% (0.9% to 7.0%)
20%	95.6%	95.2%	0.4% (0.1% to 0.7%)	16.6%	8.9%	7.7% (2.2% to 11.9%)	16.7%	8.8%	7.8% (1.6% to 12.0%)

Confidence intervals estimated using 10 000 bootstrap replications. Abbreviations: PPV, positive predictive value; RF, random forest; LR, logistic regression

[a] Binary predictions are obtained from continuous risk scores by classifying this highest-risk percentage as positive.

©2020 Potash E et al. *JAMA Network Open*.

eTable 14. Elevated Blood Lead Level Risk in 2013 Cohort by Enrollment in Women, Infants, and Children (WIC) Program

WIC Enrollment	Population incidence	Risk Ratio (95% CI)
Yes	271/5549 (4.9%)	1.4 (1.2 to 1.6)
No	679/19 383 (3.5%)	Reference

Elevated Blood Lead Level defined as venous or capillary ≥ 6µg/dL. Abbreviations: WIC, Women, Infants and Children

©2020 Potash E et al. *JAMA Network Open*.

eTable 15. Elevated Blood Lead Level Risk in 2013 Cohort by Race/Ethnicity

**Group**	**Incidence**
All	271/5549 (4.9%)
Race/ethnicity	
Hispanic	124/2637 (4.7%)
Non-Hispanic Black	131/2290 (5.7%)
Non-Hispanic White	9/274 (3.2%)
Asian	7/320 (2.2%)
Other	0/28 (0%)

©2020 Potash E et al. *JAMA Network Open*.

eTable 16. Race/Ethnicity by Risk Group for the Random Forest Model

	Race/Ethnicity				
	Hispanic	Non-Hispanic Black	Non-Hispanic White	Asian	Other
**Highest-risk %[a]**					
5%	25.5%	71.6%	2.9%	0.0%	0.0%
10%	29.9%	67.2%	2.3%	0.4%	0.1%
20%	34.6%	60.3%	2.3%	2.1%	0.7%
100%	44.9%	41.2%	6.7%	6.5%	0.7%

[a] Binary predictions are obtained from continuous risk scores by classifying this highest-risk percentage as positive.

©2020 Potash E et al. *JAMA Network Open*.

eTable 17. Confusion Matrix Metrics for the Random Forest Model by Race/Ethnicity

Specificity

	All	Race/Ethnicity			
		Hispanic	Non-Hispanic Black	Non-Hispanic White	Asian
**Highest-risk %[a]**					
5%	95.5%	97.1%	92.6%	97.4%	100.0%
10%	90.4%	93.3%	84.8%	97.0%	99.0%
20%	80.3%	84.3%	72.0%	94.0%	93.3%

Sensitivity

	All	Race/Ethnicity			
		Hispanic	Non-Hispanic Black	Non-Hispanic White	Asian
**Highest-risk %[a]**					
5%	16.2%	7.3%	26.0%	11.1%	0.0%
10%	27.3%	16.1%	38.9%	33.3%	0.0%
20%	42.4%	27.4%	58.8%	33.3%	14.3%

Positive Predictive Value

	All	Race/Ethnicity			
		Hispanic	Non-Hispanic Black	Non-Hispanic White	Asian
**Highest-risk %[a]**					
5%	15.5%	11.0%	17.5%	12.5%	NA
10%	12.7%	10.6%	13.5%	27.3%	0.0%
20%	9.9%	7.9%	11.3%	15.8%	4.5%

[a] Binary predictions are obtained from continuous risk scores by classifying this highest-risk percentage as positive.

©2020 Potash E et al. *JAMA Network Open*.

eMethods. Sensitivity Analysis

We performed a sensitivity analysis in which missing outcomes were imputed as follows:

$$P(Y_i = 1 \mid M_i = 1, X_i) = P(Y_i = 1 \mid M_i = 0, X_i) \cdot (1 + \delta)$$

Where Y_i is the binary EBLL outcome for individual i, M_i is a binary indicator for missingness of Y_i, X_i are the covariates of individual i, and δ is a sensitivity parameter so that $1 + \delta$ is the risk ratio of missing to observed outcomes. We considered $\delta = \pm 0.25$, i.e. individuals with missing outcomes being 25% more or less likely to have an EBLL than those with measured outcomes conditional on covariates. The imputation was performed in each bootstrap replication when calculating area under the receiver operating characteristic curve and confusion matrix metrics.

To estimate $P(Y_i = 1 \mid M_i = 0, X_i)$ we needed a model of the EBLL outcome. Our choice reflects two aims. First, because our task is model comparison this model should not favor either of the prediction models over the other. Second, unlike the prediction models which were fit on the training set, this model should be fit on the test set itself. Thus we use the following logistic regression model fit on the test set with available outcomes:

$$P(Y_i = 1 \mid M_i = 0, X_i) = logit^{-1}(\beta_0 + \beta_1 \hat{Y}_i^{RF} + \beta_2 \hat{Y}_i^{LR})$$

where β_j are logistic regression coefficients and $\hat{Y}_i^{RF}$, $\hat{Y}_i^{LR}$ are the (out-of-sample) random forest and logistic regression predictions for individual i with covariates X_i.

©2020 Potash E et al. *JAMA Network Open*.

eFigure. Receiver Operating Characteristic Curves by Validation Cohort

2010

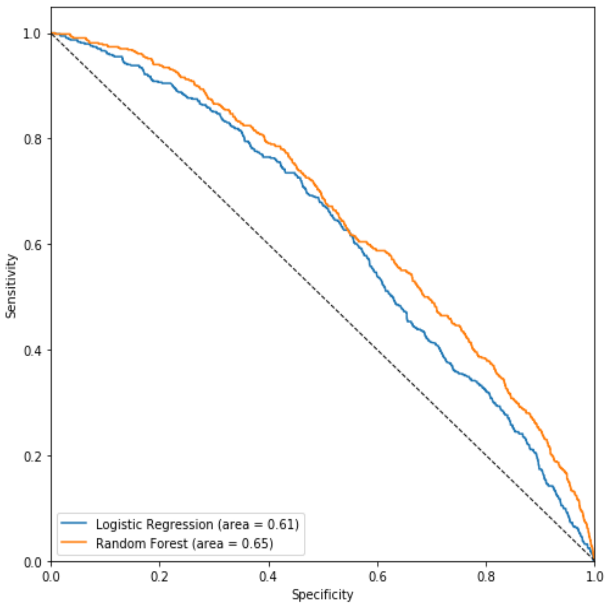

2011

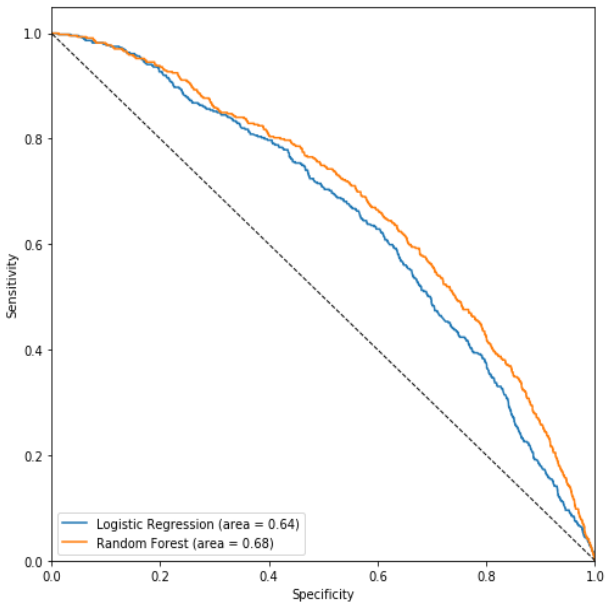

2012

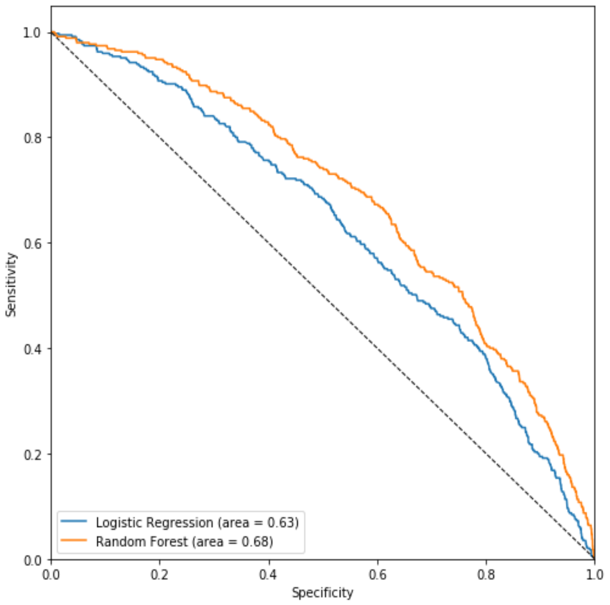

2013

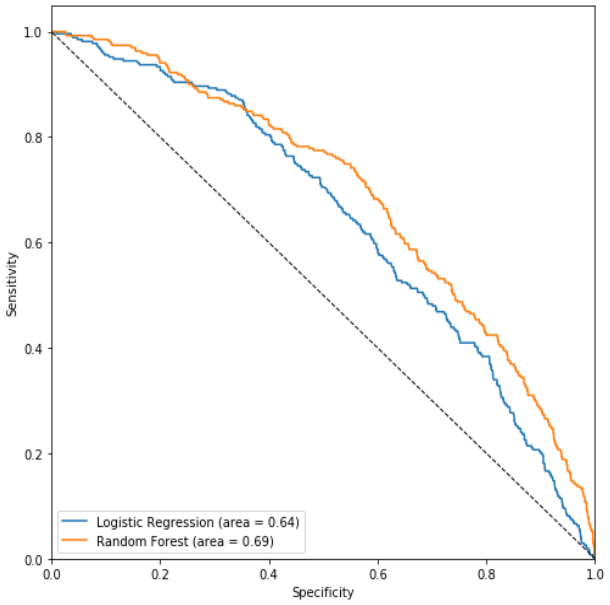

©2020 Potash E et al. *JAMA Network Open*.
